# Assessing the Initial Validity of the PortionSize App to Estimate Dietary Intake Among Adults: Pilot and Feasibility App Validation Study

**DOI:** 10.2196/38283

**Published:** 2022-06-15

**Authors:** Sanjoy Saha, Chloe Panizza Lozano, Stephanie Broyles, Corby K Martin, John W Apolzan

**Affiliations:** 1 Pennington Biomedical Research Center Louisiana State University System Baton Rouge, LA United States

**Keywords:** dietary assessment, eating, food intake, energy intake, portion size, mHealth, digital health, eHealth, nutrition, food groups

## Abstract

**Background:**

Accurately assessing dietary intake can promote improved nutrition. The PortionSize app (Pennington Biomedical Research Center) was designed to quantify and provide real-time feedback on the intake of energy, food groups, saturated fat, and added sugar.

**Objective:**

This study aimed to assess the preliminary feasibility and validity of estimating food intake via the PortionSize app among adults.

**Methods:**

A total of 15 adults (aged 18-65 years) were recruited and trained to quantify the food intake from a simulated meal by using PortionSize. Trained personnel prepared 15 simulated meals and covertly weighed (weigh back) the amount of food provided to participants as well as food waste. Equivalence tests (±25% bounds) were performed to compare PortionSize to the weigh back method.

**Results:**

Participants were aged a mean of 28 (SD 12) years, and 11 were female. The mean energy intake estimated with PortionSize was 742.9 (SD 328.2) kcal, and that estimated via weigh back was 659.3 (SD 190.7) kcal (energy intake difference: mean 83.5, SD 287.5 kcal). The methods were not equivalent in estimating energy intake (*P*=.18), and PortionSize overestimated energy intake by 83.5 kcal (12.7%) at the meal level. Estimates of portion sizes (gram weight; *P*=.01), total sugar (*P*=.049), fruit servings (*P*=.01), and dairy servings (*P*=.047) from PortionSize were equivalent to those estimated via weigh back. PortionSize was not equivalent to weigh back with regard to estimates for carbohydrate (*P*=.10), fat (*P*=.32), vegetable (*P*=.37), grain (*P*=.31), and protein servings (*P*=.87).

**Conclusions:**

Due to power limitations, the equivalence tests had large equivalence bounds. Though preliminary, the results of this small pilot study warrant the further adaptation, development, and validation of PortionSize as a means to estimate energy intake and provide users with real-time and actionable dietary feedback.

## Introduction

Accurately quantifying food intake is important to managing body weight; improving health and nutrition; and reducing the risk of chronic diseases, such as malnutrition, diabetes, and obesity [1-4]. Valid food intake assessment methods are needed to determine whether individual dietary patterns and nutrient intake meet the recommended levels [1-6]. Moreover, sufficiently accurate methods are needed that can provide people with information in real time about what foods they select and eat to facilitate the modification of dietary behaviors when they occur [1,7-10].

Traditional dietary intake assessment methods, including 24-hour dietary recall, food frequency questionnaires, and food records, have been widely used in nutritional research. These conventional methods have some advantages, as well as limitations. Recall-based methods rely on participants’ memory to recall what foods were consumed, how they were prepared, and how much of each food they consumed (ie, participants must estimate portion size). These traditional methods of dietary assessment are also time consuming [1,10,11]. Advancements in technology allow for unique perspectives when estimating energy and nutrient intake [10]. Web and food photography or food image–based methods may reduce user burden and provide more accurate estimates of food intake [10,12]. Although some of these methods may be accurate [13-15], they also have limitations. For example, food photography–based methods, such as the remote food photography method (RFPM), require trained human raters to analyze food images and quantify food intake. Consequently, such methods do not provide immediate feedback about food intake to the users, are not scalable, and have little to no cost advantage over more traditional methods [3,13,16].

PortionSize (Pennington Biomedical Research Center [PBRC]) is a newly developed smartphone app and method for estimating food intake that also relies on images of foods. Rather than human raters estimating portion size based on food images, the app integrates templates and other techniques that allow users to estimate portion sizes in real time [7]. Consequently, the users receive immediate feedback about their food selections prior to eating, which theoretically allows users to modify their food selections to better adhere to certain energy intake levels, food group recommendations, or macronutrient levels. The users also receive information about their food intake after they eat. The information provided to the users includes energy intake; fruit, vegetable, grain, protein, and dairy servings; and amounts of saturated fat and added sugar. The users also receive feedback about the extent to which their intake throughout the day is meeting specific energy intake and food group goals (eg, United States Department of Agriculture [USDA] MyPlate–recommended food groups: fruits, vegetables, grains, protein, and dairy). The results of the users estimating portion size and the ability of the users to receive food intake information immediately are expected reductions in validity and accuracy, particularly when compared to those of the RFPM [3,7,13]. To our knowledge, PortionSize is one of the first apps to provide users with food intake adherence data about their food selections before meals are consumed, after meals, and cumulatively throughout the day. We expect that the PortionSize app will help users overcome the limitations inherent with the RFPM.

**Figure 1:**
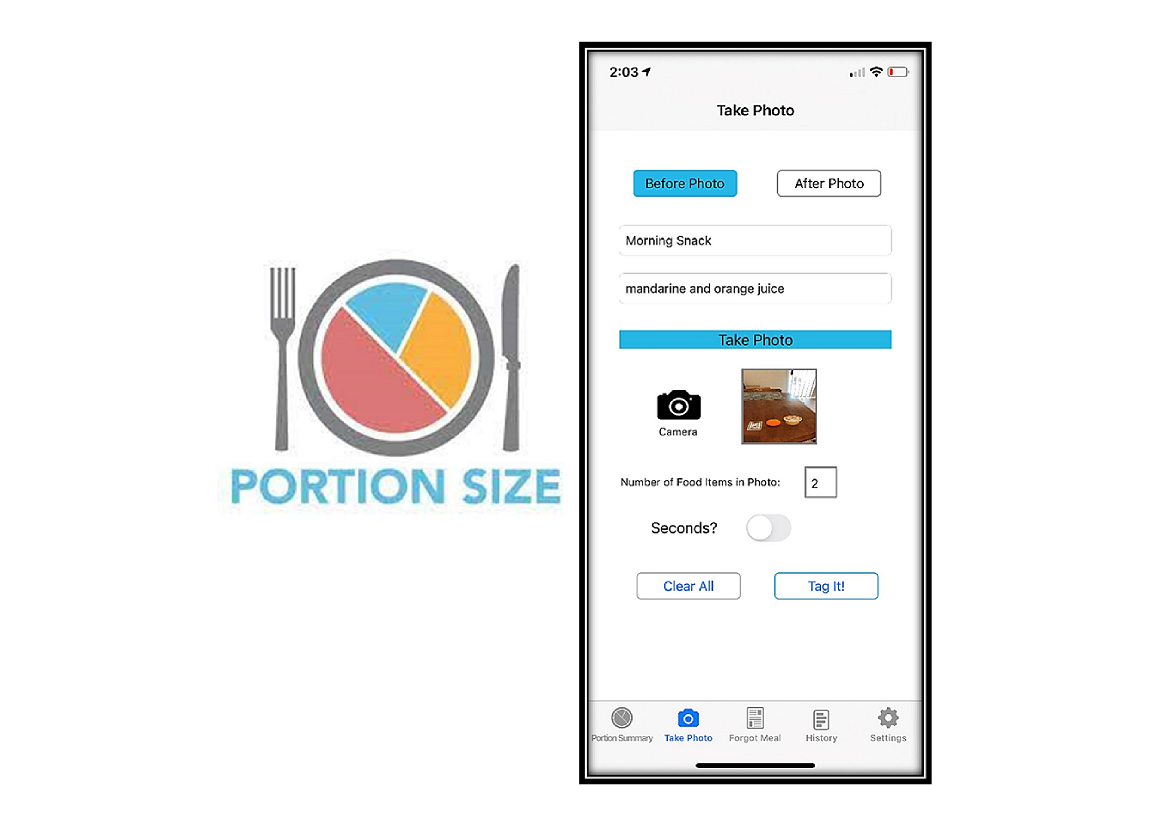
The PortionSize app allows users to take before-meal photos and after-meal photos.

The portion sizes of food have increased in the United States, and at fast-food chains, portions have increased by 2 to 5 times the original serving size [17]. Without visual aids however, it is very difficult for people [18], including trained registered dietitians [19], to accurately estimate portion sizes. Nevertheless, most dietary intake assessment methods focus on energy and nutrient intake and are not able to capture information on whether consumers are meeting the USDA MyPlate–recommended daily servings [20]. Therefore, there remains a significant need for methods that are sufficiently accurate to provide researchers with good outcome data and guide health promotion efforts while remaining scalable and affordable. PortionSize relies on emerging technology (eg, augmented reality) to improve accuracy and minimize the amount of missing data. PortionSize integrates ecological momentary assessment methods [21] to drive data completeness and quality (Figure 1). Validation studies of food intake assessment methods play an important role in identifying key areas to improve the accuracy of food intake estimation [18]. To collect preliminary data, assess initial validity, and identify areas of improvement for the PortionSize app, we conducted this pilot study. The aim of this pilot and feasibility study was to collect preliminary validity data on food intake that are estimated with the PortionSize app and compare them with data on weighed food. The secondary aim was to explore participants’ perceived satisfaction with the PortionSize app.

## Methods

### Ethics Approval

This study was conducted in accordance with the Declaration of Helsinki [22], and all procedures involving human subjects were approved by the institutional review board (IRB) at the PBRC (IRB Federal Wide Assurance number: 00006218). The trial was registered at ClinicalTrails.gov (trial number: NCT04494971) prior to recruiting participants in this study. Written informed consent was obtained from all subjects.

### Recruitment and Participants

In this pilot study, 15 adult participants were enrolled, following the recommendation made by Hertzog [23] that 10 to 15 participants are sufficient for a pilot study. Advertisements on the PBRC Current Research Trials web page and the PBRC Facebook pages were used to recruit participants. We also distributed flyers at Louisiana State University. Participants who were interested in this study emailed the study team and then completed a phone screen. Preliminary eligible participants were scheduled for an in-person screening visit. Those who qualified and remained interested enrolled in the study. The eligibility criteria were adults aged 18 to 65 years and a BMI within the range of 18.5 to 45 kg/m^2^. Participants who reported an eating disorder or serious mental illness, pregnant women, and breastfeeding mothers were excluded from this study. The aim of this pilot and feasibility study was to assess the PortionSize app’s performance when participants ate typical meals; hence, these exclusion criteria eliminated participants whose eating patterns or meal sizes may have been atypical. Each participant was given a subject ID number to ensure confidentiality, and collected data were stored in the password-protected PBRC server. The app is Health Insurance Portability and Accountability Act compliant. Participants were compensated for their successful completion of the study.

### Procedures

Participants completed a demographic questionnaire, and trained research staff conducted anthropometric measurements (height and weight) of the participants. Afterward, participants were trained to use the PortionSize app to measure food intake. During training, participants practiced assessing food intake by using food models. The entire session took about 1.5 hours.

### Measures

#### Demographics and Anthropometrics

Participants’ age, race, ethnicity, sex, marital status, education level, height, and weight were collected during their visit to the center. BMI was calculated from participants’ objectively measured height and weight.

#### Directly Weighed Food Intake to Prepare Simulated Test Meals

Trained research staff prepared simulated test meals via direct observation. Such simulated meals served as the criterion measures of portion size, energy content, and food group quantity for comparisons with the food intake assessments by participants using the PortionSize app. Participants’ energy requirements were calculated by using sex-specific formulas [24]. Energy requirements were multiplied by 1.3, and the food selection for the simulated test meal included 30% of this value, which represents a typical lunch. The meals consisted of at least 3 food items and 1 calorie-containing beverage. Menus of meals were selected from a list of commonly consumed foods from a previous study [25]. Further, 3 participants were provided with the same simulated meal food menu (Table S1 in Multimedia Appendix 1); however, the meals differed in terms of portion size. Plate waste was determined at the individual food item level. This measure ranged from 0% and 100% and was right-skewed; as such, the mean plate waste was around 5% of the foods provided, which was similar to the actual plate waste from our free-living data (around 3%) [25]. Simulated food provision and plate waste were covertly weighed, and food intake was calculated by difference.

#### Food Intake Estimation Using the PortionSize App

Participants were instructed to use the PortionSize app to estimate food provision and waste. After the assessment, participants immediately obtained estimated feedback on their food intake, including energy intake, servings of different food groups (fruits, vegetables, grains, dairy, and protein), and amounts of selected nutrients (saturated fat and added sugar). The PortionSize app currently contains a database of around 1150 food items that are linked with the food codes in the Food and Nutrition Dataset for Dietary Studies (FNDDS) database [26]. Participants identified food items (from the served simulated meals) and associated food codes by selecting food items within the PortionSize app. A summary of details about the PortionSize app are included in the supplementary materials (Multimedia Appendix 1).

#### User Satisfaction Survey and the Computer System Usability Questionnaire

After completing the food intake assessments, participants completed 2 surveys. We adapted a 10-item user satisfaction survey that was administered in prior studies to quantify satisfaction, ease of use, and the adequacy of training for PortionSize [27,28]. The items were generated to obtain user satisfaction data and feedback about the app that could be used to identify areas where the app requires improvement. We did not rely on a formal framework when developing the survey. All items were rated on a scale ranging from 1 to 6, with 1 indicating “extremely dissatisfied,” “very difficult,” “not at all,” or “not appropriate” and 6 indicating “extremely satisfied,” “extremely easy,” “very much,” or “very appropriate.”

Participants also completed the Computer System Usability Questionnaire (CSUQ; a 7-point rating scale)—a standardized, reliable, and valid questionnaire that was originally designed to evaluate computer programs [27,29]. It has been used to quantify the usability of mobile phone apps [30,31]. A rating of 7 represented “strongly disagree,” and a rating of 1 indicated “strongly agree.” The CSUQ provides an overall satisfaction score and scores for system usefulness, information quality, and interface quality [29].

### Data Analysis

All statistical analyses were performed by using IBM SPSS software (version 28.0.1; IBM Corporation) and SAS/STAT software (version 9.4; SAS Institute Inc). The primary analysis was assessing the equivalence between the PortionSize app and the weigh back method by using equivalence tests, specifically the two one-sided *t* test method [32]. The primary outcome variable was the measured energy (kcal) calculated via the weigh back method at the meal level. The equivalence bounds were set at ±25%. These bounds are large, but they reflected the appropriate statistical power for a pilot study and were used in a similar pilot study [15]. A Bland-Altman analysis [33] was performed to test for differences in error variance over levels of the variable being measured (eg, food intake). We also calculated error from PortionSize in relation to the criterion measure (weigh back) by using 2-tailed dependent samples *t* tests to compare portion sizes and the intake of energy, food group servings (fruits, vegetables, grains, dairy, and protein), macronutrients (carbohydrates, fat, and protein), selected nutrients (saturated fat, cholesterol, dietary fiber, total sugar, and added sugar), and selected micronutrients (sodium, calcium, iron, potassium, and vitamin D). These results are presented in the supplementary materials (Multimedia Appendix 1). The inclusion of selected nutrients for analysis was determined based upon the nutrition facts panels. We estimated the mean percent difference (ie, [(PortionSize − weigh back)/weigh back] × 100) at the group level to avoid having a 0 value as a denominator for each nutrient. The significance level was set at .05. User satisfaction and CSUQ survey results were presented primarily as frequencies and percentages.

## Results

### Participants’ Characteristics

A total of 21 participants completed the phone screening, and 15 participants were enrolled and completed the study. Of the 15 participants, 11 (73%) were female (Table 1). The mean age of the participants was 28 (SD 12) years, and the BMI (kg/m^2^) range was 18.8 to 41.8 kg/m^2^.

**Table 1:** Background characteristics of participants (N=15).

Variables		Value
**Sex, n (%)**		
	Male	4 (27)
	Female	11 (73)
**Race and ethnicity, n (%)**		
	Black or African American	1 (7)
	White	14 (93)
**Education, n (%)**		
	High school diploma or General Educational Development	1 (7)
	Some college	7 (47)
	Bachelor’s degree	5 (33)
	Postgraduate degree	2 (13)
**Employment, n (%)**		
	Unemployed	2 (13)
	Full-time employment	4 (27)
	Part-time employment	7 (47)
	Retired	1 (7)
	Other: student	1 (7)
Age (years), mean (SD; range)		28.0 (12.2; 20-57)
Height (cm), mean (SD; range)		168.1 (10.4; 147.3-182.9)
Weight (kg), mean (SD; range)		68.3 (19.8; 50.4-113.4)
BMI (kg/m^2^), mean (SD; range)		24.1 (6.6; 18.8-41.8)

### Estimation of Energy Intake

**Figure 2:**
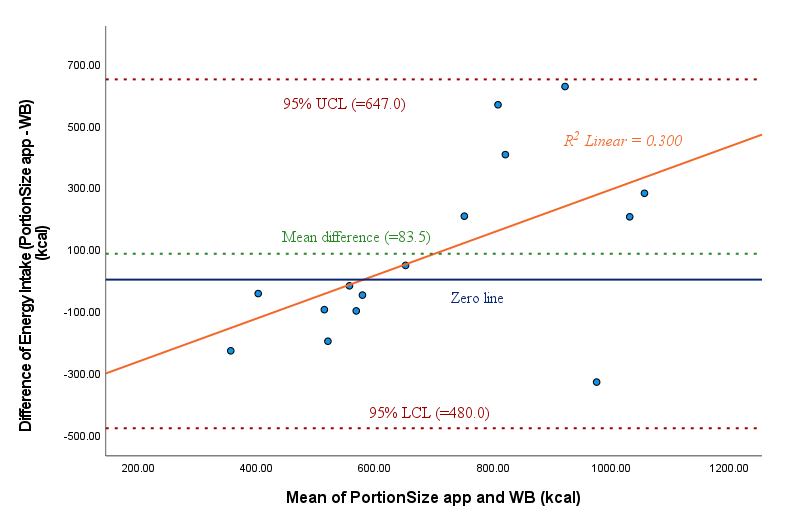
Bland-Altman analysis for comparing energy intake (kcal) between PortionSize and the WB method (15 meals). LCL: lower confidence limit; UCL: upper confidence limit; WB: weigh back.

Table 2 indicates that the mean energy intake estimated with the PortionSize app (742.9, SD 328.2 kcal) was not equivalent (*P*=.18) to the mean estimated from the weighed meals (659.3, SD 190.7 kcal). The mean energy intake difference between the two methods was 83.5 (95% CI –480.0 to 647.0) kcal, and the mean percent error for the estimation of energy intake was 12.7% (Table 2 and Table S2 in Multimedia Appendix 1). PortionSize underestimated energy intake at lower levels of intake, but overestimation occurred and increased with higher levels of intake (Figure 2), as indicated by a significant regression equation (*R*^2^=0.300; adjusted *R*^2^=0.246; *P*=.03).

**Table 2:** Comparison of portion size, energy, and nutrient intake estimates between PortionSize and the weigh back method (meals: N=15).

	PortionSize app			Weigh back			Difference			Equivalence at ±25%, *P* value		Mean percent error^a^
	Mean	SD	Mean		SD	Mean		SD				
Energy (kcal)	742.9	328.2	659.3		190.7	83.5		287.5	.18		12.7	
Portion size (g)	674.3	222.8	716.9		207.2	−42.7		303.9	.03^b^		−6	
Total fruits (servings^c^)	0.2	0.3	0.3		0.4	−0.1		0.4	.01^b^		−33.3	
Total vegetables (servings^c^)	0.6	0.3	0.6		0.4	0.0		0.2	.37		0	
Total grains (servings^d^)	1.7	1.7	1.2		1.1	0.5		0.8	.31		41.7	
Total dairy (servings^c^)	0.4	0.6	0.5		0.6	−0.1		0.7	.047^b^		−20	
Total protein (servings^d^)	3.1	3.6	2.8		2.9	0.3		2.0	.87		10.7	
Saturated fat (g)	10.8	6.8	10.4		4.1	0.4		6.1	.11		3.8	
Added sugar (teaspoons)	8.8	7.3	8.5		5.1	0.2		4.8	.14		2.4	
Protein (g)	35.3	27.9	32.7		19.8	2.6		17.4	.25		8	
Total fat (g)	32.5	22.4	27.1		10.8	5.4		18.7	.32		19.9	
Carbohydrates (g)	78.9	49.6	72.0		28.4	6.9		33.8	.10		9.6	
Dietary fiber (g)	4.6	3.2	4.1		1.3	0.5		3.6	.39		12.2	
Total sugar (g)	45.6	27.6	46.5		15.7	−1.0		23.7	.049^b^		−2.2	
Cholesterol (mg)	110.3	94.0	103.3		73.0	7.1		61.9	.52		6.9	
Sodium (mg)	1200.8	562.0	940.5		376.7	260.3		449.5	.68		27.7	
Calcium (mg)	214.3	185.4	254.7		176.5	−40.4		171.7	.09		−15.9	
Iron (mg)	4.3	3.1	3.3		2.3	1.0		1.4	.72		30.3	
Potassium (mg)	888.5	576.6	831.9		431.8	56.6		361.1	.049^b^		6.8	
Vitamin D (µg)	0.8	1.9	1.5		2.0	−0.6		2.1	.62		−40	

^a^Mean percent error = ([PortionSize – weigh back]/weigh back) × 100.

^b^Significant equivalence (level of significance at *P*<.05).

^c^Servings were cup equivalents.

^d^Servings were ounce equivalents.

### Estimation of Portion Size

**Figure 3:**
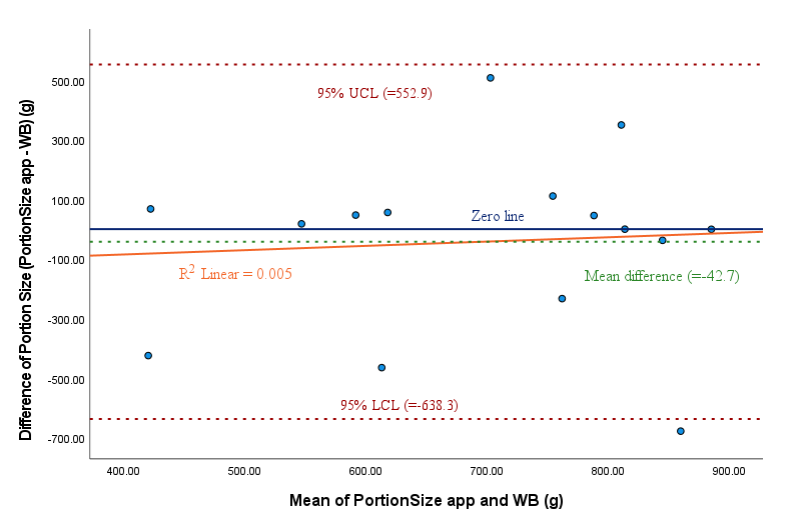
Bland-Altman analysis for comparing consumed food in grams (portion size) between PortionSize and the WB method (15 meals). LCL: lower confidence limit; UCL: upper confidence limit; WB: weigh back.

The mean gram weight of meals estimated with the PortionSize app was 674.3 (SD 222.8) g, and the mean estimated from the weighed meals was 716.9 (SD 207.2) g. The mean difference in the estimated gram weights of food items between the two methods was −42.7 (95% CI −638.3 to 552.9) g/meal, and both means were significantly equivalent (*P*=.03; Table 2 and Table S2 in Multimedia Appendix 1). The mean percent error for the estimation of gram weight was −6%. The Bland-Altman limit of agreement plot for the gram weights of food items (Figure 3) indicates a slightly positive trend (not significant) for the difference in estimated gram weights (PortionSize – weigh back) with regard to the means of both methods ([PortionSize + weigh back]/2). The results show nonsignificant bias (*R*^2^=.005; adjusted *R*^2^=−0.071; *P*=.80).

### Estimation of Food Group Servings

The mean PortionSize-estimated total fruit servings was 0.2 (SD 0.3; Table 2). The mean weigh back method–estimated servings of fruits was 0.3 (SD 0.4), and the means were equivalent (*P*=.01). PortionSize-estimated total dairy servings (mean 0.4, SD 0.6 servings) were equivalent to the weigh back–estimated servings (mean 0.5, SD 0.6 servings; *P*=.047). Among the five food groups, the estimations of total vegetable servings had the lowest mean percent error (0%), with those for grain servings having the highest (41.7%). The results of dependent *t* tests for comparing the intake of energy, nutrient, and food group servings between the two methods—the PortionSize app and weigh back—are presented in Table S2 in Multimedia Appendix 1. Bland-Altman analyses of food groups are presented in Figures S4-S8 in Multimedia Appendix 1.

### Estimation of Macronutrients and Specific Nutrients

The estimates for the mean intake of protein (*P*=.25), fat (*P*=.32), and carbohydrates (*P*=.10) were not equivalent between PortionSize and the weigh back method (Table 2). The mean differences in protein, fat, and carbohydrate intake estimates between PortionSize and the weigh back method were 2.6 (SD 17.4) g, 5.4 (SD 18.7) g, and 6.9 (SD 33.8) g, respectively. We found significant equivalence in estimations of total sugar (*P*=.049) and potassium (*P*=.049) intake between PortionSize and the weigh back method. Among the macronutrient estimations, total fat estimations had the highest mean percent error (19.9%).

### User Satisfaction and CSUQ Survey

Table 3 shows that of the 15 participants, 12 (80%) were satisfied or extremely satisfied with the PortionSize app, and 11 (73%) were similarly satisfied with the easiness of the PortionSize app for recording portion sizes. Moreover, 13 (87%) participants marked “very much” for how much the iPhone training helped them to prepare for using the PortionSize app.

The CSUQ survey indicated that 11 (73%) participants strongly agreed that they could become productive quickly by using the app, and the information provided for the app was easy to understand (Table S3 in Multimedia Appendix 1). The mean score from the CSUQ was 35.3 (SD 13.5).

**Table 3:** Participants’ satisfaction with the PortionSize app (N=15).

Questions	Score, n (%)					
	1^a^	2	3	4	5	6^b^
1. How satisfied are you with the PortionSize app for recording portion sizes?	N/A^c^	N/A	N/A	3 (20)	8 (53)	4 (27)
2. How satisfied are you with the *Portion Summary* tab of the PortionSize app?	N/A	N/A	2 (13)	1 (7)	6 (40)	6 (40)
3. How satisfied are you with the *Take Photo* tab of the PortionSize app?	N/A	N/A	N/A	4 (27)	6 (40)	5 (33)
4. How satisfied are you with the *Forgot Meal* tab of the PortionSize app?	N/A	1 (7)	N/A	N/A	4 (27)	10 (67)
5. How satisfied are you with the *Settings* tab of the PortionSize app?	N/A	N/A	N/A	1 (7)	4 (27)	10 (67)
6. How easy was it to use the PortionSize app for recording portion sizes?	N/A	N/A	N/A	4 (27)	7 (47)	4 (27)
7. How easy was it to capture images and record portion sizes?	N/A	N/A	1 (7)	7 (47)	4 (27)	3 (20)
8. How easy was it to use the *Forgot Meal* tab to describe portions?	N/A	N/A	2 (13)	1 (7)	4 (27)	8 (53)
9. How much did the iPhone training help prepare you for using the PortionSize app?	N/A	N/A	N/A	N/A	2 (13)	13 (87)
10. How appropriate were the PortionSize templates superimposed on your food items?	N/A	N/A	N/A	1 (7)	8 (53)	6 (40)

^a^Scores of 1 indicated “extremely dissatisfied,” “very difficult,” “not at all,” and “not appropriate” for questions 1 to 5, questions 6 to 8, question 9, and question 10, respectively.

^b^Scores of 6 indicated “extremely satisfied,” “extremely easy,” “very much,” and “very appropriate” for questions 1 to 5, questions 6 to 8, question 9, and question 10, respectively.

^c^N/A: not applicable.

## Discussion

### Principal Findings

In this small pilot and feasibility study, we collected preliminary validation data for the PortionSize app. The results indicate that the estimations of energy intake and the intake of energy-contributing nutrients (carbohydrates, protein, and fat) from the PortionSize app were not equivalent to those estimated via weigh back. The mean percent error of the energy intakes estimated by the PortionSize app and from weighed food was 12.7% and fell within the ranges of 8% to 30% for 24-hour dietary recall and 1.3% to 47% for diet histories, food records, and food frequency questionnaires [1]. PortionSize’s error for estimating gram weight intake was smaller (−6%). There are mixed results that suggest that app-based assessment methods either underestimate or overestimate energy intake when compared with the doubly labeled water method and traditional methods, such as dietary records [1,9]. Traditional methods, such as self-reported 24-hour dietary recall, have significantly underreported energy intake when compared with 7-day food weigh records [34]. A systematic review and meta-analysis study found that image-based dietary assessments underestimated energy intake by 20% (range 0%-37%) when compared with the doubly labeled water method; however, the study showed no significant difference in energy intakes estimated via traditional methods (such as 24-hour dietary recall) and the RFPM [35]. We observed in this pilot study that the CI for the mean difference in energy intake estimations between the two methods—the PortionSize app and weigh back—crossed 0 (Table S2 in Multimedia Appendix 1).

The results of this pilot study indicate that the estimated portion sizes (g) of food from the PortionSize app was equivalent to the portion sizes that were estimated via weigh back, and the mean percent error between the two methods was −6%. Inaccurate portion size estimates necessarily result in inaccurate food intake estimates, and approximately 50% of the error in self-reported food intake is due to inaccurate portion size estimates, with missing data likely accounting for the majority of the remaining error [36]. Furthermore, consumers’ difficulties with estimating portion sizes are a barrier to correctly measuring energy intake [37]. The PortionSize app integrates visual templates that enable users or consumers to accurately quantify portion sizes in real time [7]. However, users need to be discreet when selecting the right FNDDS food codes within the PortionSize app to match with the food items in order to correctly estimate their food intake.

Equivalent estimations of total fruit and total dairy servings were found between PortionSize and the weigh back method. We cross-checked the outlier values for the vegetable, grain, and protein group servings. A participant experienced app glitches when using the PortionSize app and thus could not correctly record plate waste for the grain group servings (outlier value is reported in Figure S6 in Multimedia Appendix 1). In addition, 1 participant mistakenly reported percent plate waste when using the PortionSize app and thus generated an outlier value for the protein group servings (Figure S8 in Multimedia Appendix 1). The estimations of total vegetable servings were not equivalent; however, the mean percent error was 0%. This reflects that there is no fundamental issue with the PortionSize app; however, app improvements are needed for the correct estimation of food intake.

The USDA provides recommendations in terms of food groups, such as fruits, vegetables, grains, protein, and dairy [20]. To our knowledge, PortionSize app is the first food intake assessment tool that provides immediate feedback on energy intake and food group servings. Such feedback can help users track whether they are meeting the recommended daily intake of energy, fruits, vegetables, grains, dairy, and protein. Additionally, obtaining real-time feedback on food selection will provide an opportunity for users to modify their food intake and thus improve their food intake behavior [3,38,39]. Unspecific or delayed feedback is not as effective as real-time feedback at inducing behavior change, since behavior change is promoted by receiving immediate and specific feedback based on objective data that are temporally associated with a target behavior [38]. Food intake and dietary patterns outline an individual’s nutrition status because food intake encompasses energy intake; nutrient intake (macronutrients and micronutrients, including vitamins and minerals); and the consumption of different food groups, such as fruits and vegetables [1,17,40].

A survey-based study that focused on the perceived burdens of and preferences for traditional methods, the RFPM, and PortionSize indicated that 67.3% of participants preferred to use the RFPM, 51.9% preferred the PortionSize app, 48% preferred food records, and 32.9% preferred 24-hour dietary recall. Nevertheless, a significantly higher percentage of older adults (aged ≥65 years) preferred using food records and 24-dietary recall when compared to other participants (aged <65 years) [7]. Older adults often perceive barriers and difficulties in using mobile health apps [41], and this could be one of the reasons that older adults typically prefer traditional methods. Participants in the survey-based study perceived the RFPM to less burdensome compared to the PortionSize app [7]; however, the RFPM needs trained human raters to analyze food images and assess food intake [16]. On the other hand, the PortionSize app has more embedded features for measuring food intake, including those for capturing images of food selection and plate waste, identifying foods, and estimating portion size, and these may challenge users or consumers when using the PortionSize app. Low health and nutrition literacy could be potential barriers to accurately estimating portion size [40]. The PortionSize app provides real-time feedback on food intake and food group servings; therefore, we expect that the advantages of using PortionSize app will promote users’ willingness to use the PortionSize app and accept the challenges. We also expect that future validation studies will support this hypothesis.

### Limitations

The purpose of this pilot and feasibility study was to examine the initial validity of the PortionSize app and inform power for future validation studies of the PortionSize app. This study has several limitations that need to be acknowledged. First, it had a small but appropriate sample size for a pilot study. Second, most of the participants were female (11/15, 73%) and highly educated (college or above: 14/15, 93%). Third, there was limited representation from different racial or ethnic groups. Fourth, while all BMI categories were targeted, the effects across different BMI categories could not be examined due to the small sample size. Fifth, each participant estimated a single meal with limited food items; however, they did not estimate food intake over a long duration (ie, days or weeks). Sixth, the PortionSize app was also designed to estimate and provide feedback on alcohol consumption; however, we did not analyze alcohol consumption in this study. Lastly, the PortionSize app was recently developed, and the team continues to debug the app and improve its functionality. This likely impacted user satisfaction. Future studies with a large sample are needed to examine differences in food intake estimations from the PortionSize app between men and women, among BMI categories, and among different ethnic groups.

### Conclusions

The findings from this pilot study suggest that the PortionSize app has promise for estimating food intake in real time. With some improvements, it is hoped that the PortionSize app will become sufficiently accurate, so that it can be used by participants to modify their food intake in real time (ie, when they are selecting foods) and how much of each food they eat during a meal or snack.

## Appendix

Multimedia Appendix 1 Supplementary material.

## Abbreviations

CSUQ: Computer System Usability Questionnaire
FNDDS: Food and Nutrition Dataset for Dietary Studies
IRB: institutional review board
PBRC: Pennington Biomedical Research Center
RFPM: remote food photography method
USDA: United States Department of Agriculture
