# Supplementary material for: Assessing the Initial Validity of the PortionSize App to Estimate Dietary Intake Among Adults: Pilot and Feasibility App Validation Study
Source: JMIR Form Res. 2022 Jun 15;6(6):e38283. doi: 10.2196/38283 (PMC9244674; doi:10.2196/38283)
Supplement: Multimedia Appendix 1 [file formative_v6i6e38283_app1.docx]

**Supplementary Material**

PortionSize app

PortionSize is an app that enables users to assess food selection and food consumption in real-time. As the user eats throughout the day, the app provides immediate feedback on the energy and nutrient content of meals before they are eaten. After the meal (and after the app takes into consideration plate waste), immediate feedback about food intake is provided at the meal and cumulatively at the day level (Suppl. Figure 1). The Dashboard is updated in real-time and easily allows the user to see if they have exceeded their daily energy intake or food group targets. Participants can go back to each eating occasion based on date and meal. For example, in Suppl. Figure 1, the user has not yet exceeded their calorie allotment for the day, but the user did exceed their targeted dairy and saturated fat intake. Currently, PortionSize promotes adherence to the USDA MyPlate recommendations, though other dietary approaches (e.g., low carbohydrate diets, Mediterranean diet) can be accommodated.

When using PortionSize a reference card is placed next to users’ foods when images are captured (Suppl. Figure 2). We use augmented reality to change the outline of the card in the viewfinder from red to green, at which time the distance to the card is between 1.9 to 2.1 feet (2.0 feet is the goal). The user is then prompted to capture the image. Participants identify foods in their images by ‘tagging’ them. Specifically, they tap a food and then look up the food in the database by: a) entering a search term, or b) using a drop-down menu (e.g., selecting ‘Protein,’ then selecting ‘Egg,’ ‘Egg whole,’ and ‘Cooked with fat). These selections identify the template that is used to estimate portion size (e.g., a deck of cards for steak), and the app then links those portion size estimates to the correct row of data in the onboard database to calculate food intake. A text box is also present that allows the user to enter additional information about their food and beverages and these data are immediately transmitted with the images and other data to the server of PBRC. We adapted our visual comparison methodology to create a template system that allows users to estimate the portion size of foods in their images. The semi-transparent templates can be increased or decreased in size from 0.2 to 5 times the original template by using a slider (Suppl. Figure 3). They are also moveable and can be manipulated to overlay foods in users’ images in the app (e.g., in Figure 3, the deck of cards template is used to estimate the amount of scrambled eggs in the image). The app then calculates food intake based on the size of the templates via the nutrient database in the app.


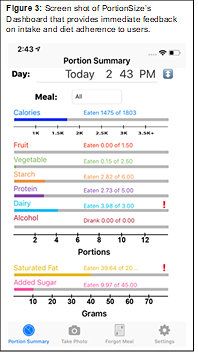


**Figure S1.** Screen shot of PortionSize’s Dashboard that provides immediate feedback on intake and diet adherence to users


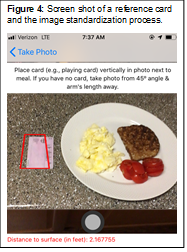


**Figure S2.** Screen shot of a reference card and the image standardization process


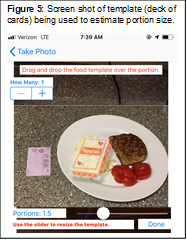


**Figure S3.** Screen shot of a template (deck of cards) being used to estimate portion size

Food intake is equal to food selection minus plate waste, and we found that in free-living conditions plate waste is very small (~3.3% of all food selected), and higher estimates from the literature are due to relying on data from buffets and atypical eating conditions, such as laboratory-based food intake tests that provide large amounts of food [25]. Based on these data and to manage user burden, plate waste is currently quantified in PortionSize by allowing the user to report that they ‘ate everything’, or to enter the percent of their foods that remained at the end of the meal.

**Table S1.** Food menus

| Menu 1: Burger Lab Meal | Menu 2: Chicken and Rice Lab Meal | Menu 3: Pizza and Salad Lab Meal | Menu 4: Pork Chop Lab Meal | Menu 5: Salad Lab Meal |
| --- | --- | --- | --- | --- |
| Burger Patties | Chicken | Pizza | Pork Chops | Lettuce |
| Tomato | Ready Rice | Lettuce Mix | Broccoli | Carrot |
| Onion | Oranges | Tomato | Butter | Tomato |
| Burger Buns | Carrots | Dressing | Apples | Pre-Cooked Chicken (Frozen) |
| Cheese Slices | Butter | Cookies | Tea | Dressing |
| Ketchup | Tea | Coke |  | Milk |
| Cookies |  |  |  |  |
| Coke |  |  |  |  |

**Table S2.** Comparison of portion size, energy, and nutrient intake estimates between PortionSize and weigh back (N = 15 meals)

|  | Difference between PortionSize and weigh back | | 95% CI  (Lower- Upper) | |
| --- | --- | --- | --- | --- |
|  | Mean | SD |  |  |
| Energy (kcal) | 83.5 | 287.5 | -75.7 | 242.7 |
| Portion Size (g) | -42.7 | 303.9 | -210.9 | 125.6 |
| Total fruit (servings) | -0.1 | 0.4 | -0.3 | 0.1 |
| Total vegetable (servings) | 0.0 | 0.2 | -0.1 | 0.2 |
| Total grain (servings) | 0.5 | 0.8 | 0.0 | 0.9 |
| Total dairy (serving) | -0.1 | 0.7 | -0.5 | 0.3 |
| Total protein (servings) | 0.3 | 2.0 | -0.9 | 1.4 |
| Saturated fat (g) | 0.4 | 6.1 | -3.0 | 3.8 |
| Added sugar (Tbs) | 0.2 | 4.8 | -2.5 | 2.9 |
| Protein (g) | 2.6 | 17.4 | -7.1 | 12.2 |
| Total Fat (g) | 5.4 | 18.7 | -4.9 | 15.7 |
| Carbohydrate (g) | 6.9 | 33.8 | -11.9 | 25.6 |
| Dietary fiber (g) | 0.5 | 3.6 | -1.5 | 2.4 |
| Total sugar (g) | -1.0 | 23.7 | -14.1 | 12.2 |
| Cholesterol (mg) | 7.1 | 61.9 | -27.2 | 41.3 |
| Sodium (mg) | 260.3 | 449.5 | 11.4 | 509.2 |
| Calcium (mg) | -40.4 | 171.7 | -135.5 | 54.7 |
| Iron (mg) | 1.0 | 1.4 | 0.2 | 1.8 |
| Potassium (mg) | 56.8 | 361.1 | -143.3 | 256.6 |
| Vitamin D (µg) | -0.6 | 2.1 | -1.8 | 0.5 |

CI: Confidence intervals; SD: Standard deviation


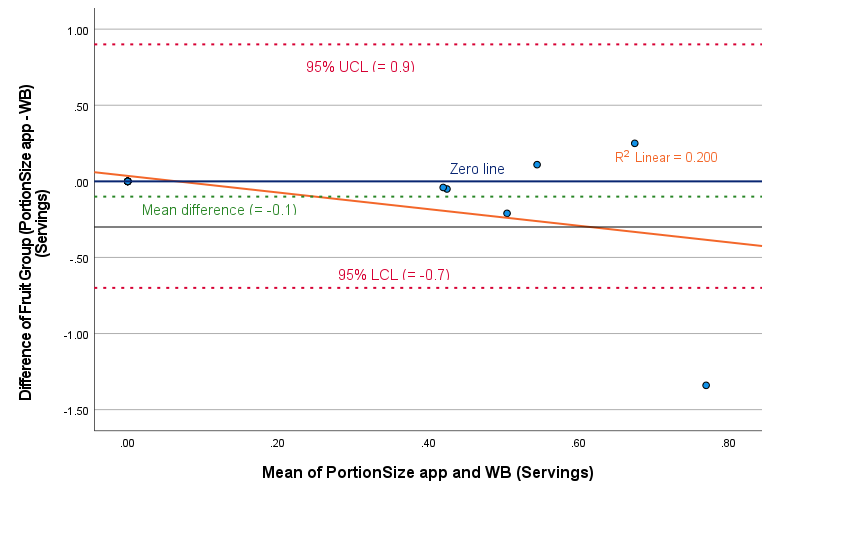


**Figure S4.** Bland and Altman analysis comparing fruit group intake (servings) between PortionSize and weigh back (15 meals)

*
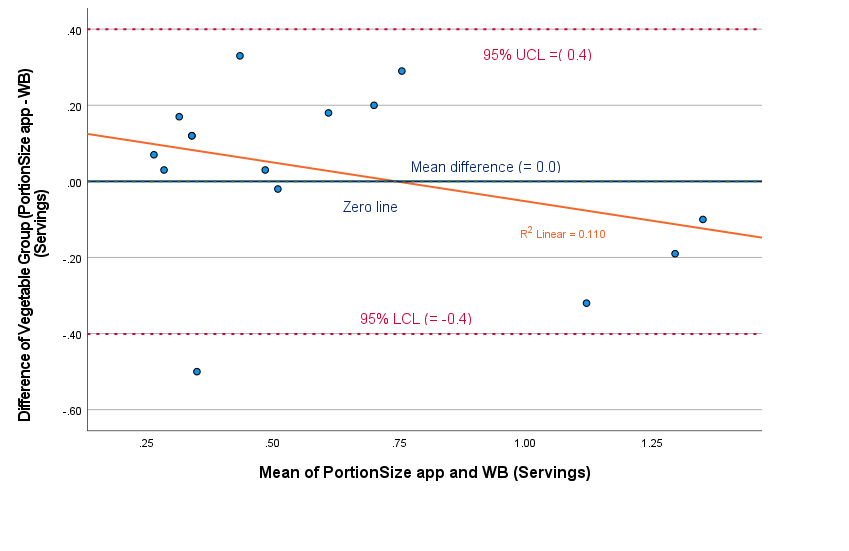
*

**Figure S5.** Bland and Altman analysis comparing vegetable group intake (servings) between PortionSize and weigh back (15 meals)


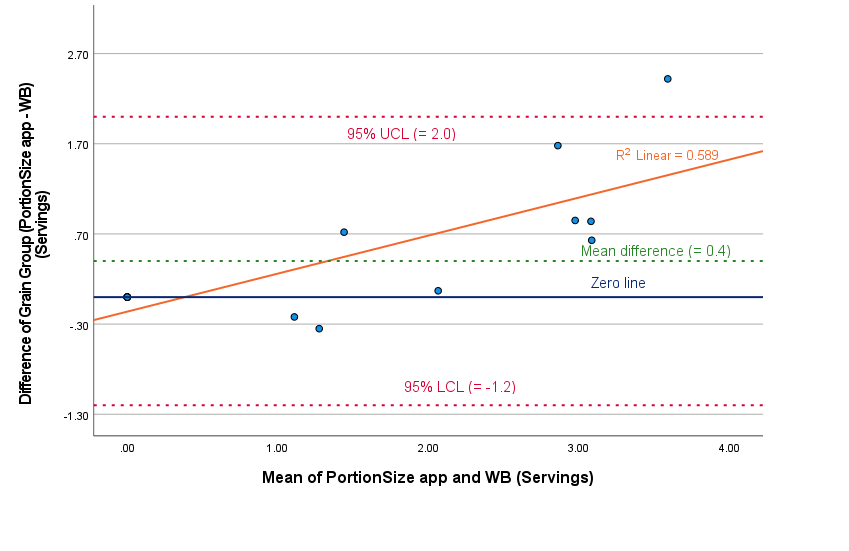


**Figure S6.** Bland and Altman analysis comparing grain group intake (servings) between PortionSize and weigh back (15 meals)


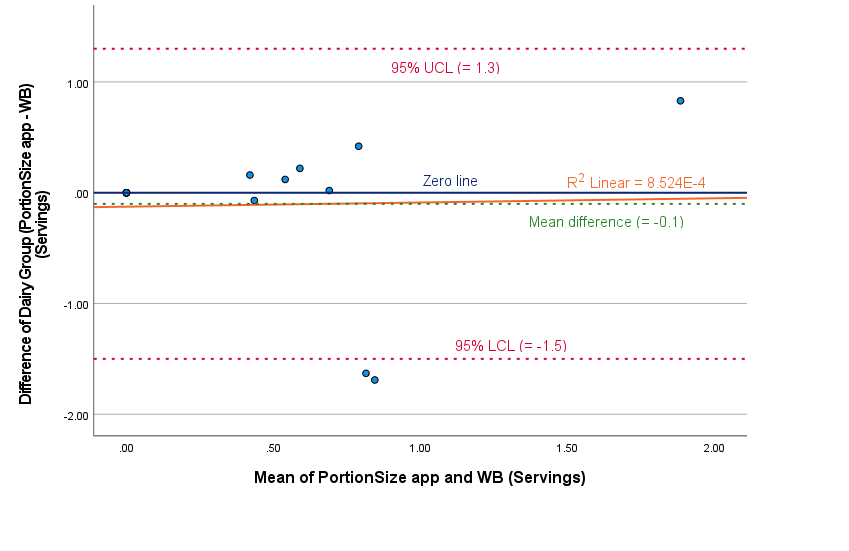
**Figure S*7*.** Bland and Altman analysis comparing dairy group intake (servings) between PortionSize and weigh back (15 meals)


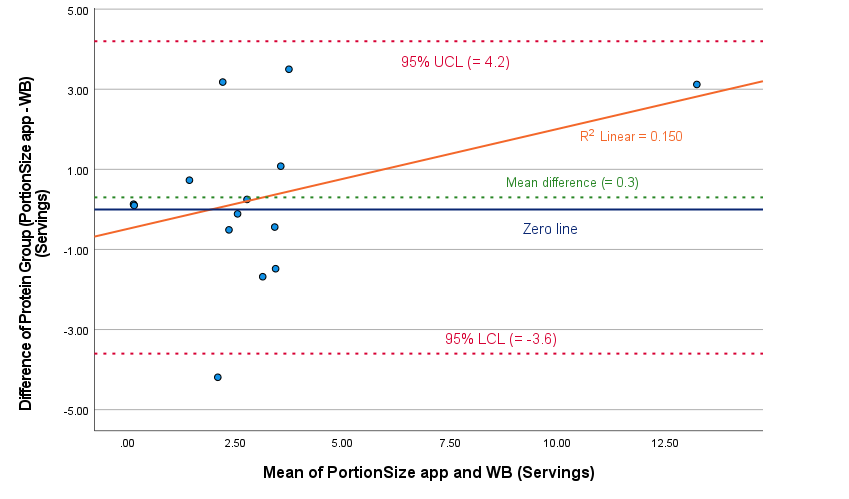


**Figure S8.** Bland and Altman analysis comparing protein group intake (servings) between PortionSize and weigh back (15 meals)

**Table S3.** Computer system usability questionnaire survey (N = 15)

|  | Questions | Strongly agree  1  n (%) | 2  n (%) | 3  n (%) | 4  n (%) | 5  n (%) | 6  n (%) | Strongly disagree  7  n (%) | N/A |
| --- | --- | --- | --- | --- | --- | --- | --- | --- | --- |
| 1 | Overall, I am satisfied with how easy it was to use the app | 4 (26.7) | 10 (66.7) | - | - | 1 (6.7) | - | - | - |
| 2 | It was simple to use the app | 5 (33.3) | 7 (46.7) | 2 (13.3) | - | 1 (6.7) | - | - | - |
| 3 | I could effectively complete the tasks using the app | 8 (53.3) | 5 (33.3) | 1 (6.7) | 1 (6.7) | - | - | - | - |
| 4 | I was able to complete the tasks quickly using the app | 2 (13.3) | 5 (33.3) | 5 (33.3) | 2 (13.3) | 1 (6.7) | - | - | - |
| 5 | I was able to efficiently complete tasks using the app | 4 (26.7) | 8 (53.3) | 3 (20.0) | - | - | - | - | - |
| 6 | I felt comfortable using the app | 7 (46.7) | 6 (40.0) | - | 2 (13.3) | - | - | - | - |
| 7 | It was easy to learn to use the app | 10 (66.7) | 4 (26.7) | - | - | 1 (6.7) | - | - | - |
| 8 | I believe I could become productive quickly using the app | 11 (73.3) | 3 (20.0) | 1 (6.7) | - | - | - | - | - |
| 9 | The app gave error messages that clearly told me how to fix the problems* | 2 (14.3) | 2 (14.3) | 2 (14.3) | 1 (7.1) | 2 (14.3) | - | - | 5 (35.7) |
| 10 | Whenever I made a mistake using the app, I could recover easily and quickly | 2 (13.3) | 6 (40.0) | 3 (20.0) | - | - | 2 (13.3) | - | 2 (13.3) |
| 11 | The information (on screen messages, handouts) provided with this app was clear | 10 (66.7) | 4 (26.7) | 1 (6.7) | - | - | - | - | - |
| 12 | It was easy to find the information I needed | 8 (53.3) | 3 (33.3) | 2 (13.3) | 2 (13.3) | - | - | - | - |
| 13 | The information provided for the app was easy to understand | 11 (73.3) | 2 (13.3) | 2 (13.3) | - | - | - | - | - |
| 14 | The information was effective in helping me complete the tasks | 9 (60.0) | 4 (26.7) | 1 (6.7) | 1 (6.7) | - | - | - | - |
| 15 | The organization of information on the app display was clear | 9 (60.0) | 3 (20.0) | 3 (20.0) | - | - | - | - | - |
| 16 | The interface of the system was pleasant | 7 (46.7) | 4 (26.7) | 1 (6.7) | 1 (6.7) | 2 (13.3) | - | - | - |
| 17 | I liked using the interface of the app | 5 (33.3) | 5 (33.3) | 2 (13.3) | 1 (6.7) | 2 (13.3) | - | - | - |
| 18 | The app has all the functions and compatibilities I expect it to have | 7 (46.7) | 3 (20.0) | 1 (6.7) | 3 (20.0) | 1 (6.7) | - | - | - |
| 19 | Overall, I am satisfied with the app | 6 (40.0) | 6 (40.0) | 2 (13.3) | 1 (6.7) | - | - | - | - |

* One missing (n = 14 participants)
